# A molecularly defined skin test reagent for the diagnosis of bovine tuberculosis compatible with vaccination against Johne’s Disease

**DOI:** 10.1038/s41598-021-82434-7

**Published:** 2021-02-03

**Authors:** Sonya Middleton, Sabine Steinbach, Michael Coad, Kevina McGill, Colm Brady, Anthony Duignan, Jimmy Wiseman, Eamonn Gormley, Gareth J. Jones, H. Martin Vordermeier

**Affiliations:** 1 Animal and Plant Health Agency, Bacteriology, Addlestone, UK; 2 University College, Dublin, Republic of Ireland; 3 Department of Agriculture Food and the Marine (DAFM), Dublin, Republic of Ireland

**Keywords:** Immunology, Infectious diseases, Peptides

## Abstract

Tuberculin Purified Protein Derivatives (PPDs) exhibit multiple limitations: they are crude extracts from mycobacterial cultures with largely unknown active components; their production depends on culture of mycobacteria requiring expensive BCL3 production facilities; and their potency depends on the technically demanding guinea pig assay. To overcome these limitations, we developed a molecularly defined tuberculin (MDT) by adding further antigens to our prototype reagent composed of ESAT-6, CFP-10 and Rv3615c (DIVA skin test, DST). In vitro screening using PBMC from infected and uninfected cattle shortlisted four antigens from a literature-based list of 18 to formulate the MDT. These four antigens plus the previously identified Rv3020c protein, produced as recombinant proteins or overlapping synthetic peptides, were formulated together with the three DST antigens into the MDT to test cattle experimentally and naturally infected with *M*. *bovis*, uninfected cattle and MAP vaccinated calves. We demonstrated significant increases in MDT-induced skin responses compared to DST in infected animals, whilst maintaining high specificity in unvaccinated or MAP vaccinated calves. Further, MDT can also be applied in in vitro blood-based interferon-gamma release assays. Thus, MDT promises to be a robust diagnostic skin and blood test reagent overcoming some of the limitations of PPDs and warrants full validation.

## Introduction

Bovine Tuberculosis (bTB) is a disease of economically important livestock species of world-wide distribution, such as cattle and goats. BTB can be caused by pathogens of the *Mycobacterium tuberculosis* group of mycobacteria including *M. tuberculosis* itself. Nevertheless, in many countries including the United Kingdom, bTB is caused almost exclusively by *M. bovis*, a pathogen with a wide host range which, in the UK includes badgers (*Meles meles*)^1,2^. BTB is an important source of economic loss, both through loss of productivity and the cost of control programmes, estimated to around US$3 billion per year worldwide^3^; in the UK the cost of controlling the disease is estimated at £120 million per year^4^. This disease has also important zoonotic consequences, particularly in many low to middle income countries (LMIC) where milk pasteurisation cannot be guaranteed. The zoonotic relevance of this disease has been recently recognised in the publication of a roadmap to control zoonotic tuberculosis, jointly published by the WHO, OIE and the Union against Tuberculosis and Lung Disease^5^.

In many high income countries bTB has been eradicated by applying a test and slaughter programme as part of an eradication strategy^6,7^. However, in a number of countries such as the United Kingdom, Ireland and Spain, bTB eradication has not yet been achieved and this persistence has often been attributed to wildlife maintenance hosts^2,8,9^. Test and slaughter strategies are conventionally based on active surveillance through the application of Purified Protein Derivatives of Tuberculin (PPD)-based skin testing, with interferon-gamma release assays (IGRAs) often applied as ancillary tests to maximise the detection of infected cattle. Various forms of the tuberculin skin test are employed to test cattle. In many countries, incuding the UK, Ireland, France and Portugal, the routine surveillance screening test for bTB in cattle is the Comparative Cervical Tuberculin (CCT) test, which requires the injection of PPDs prepared from *M. avium* (avian PPD = PPD-A) and from *M. bovis* (bovine PPD = PPD-B). The cut-off applied predominantly in screening protocols is a difference of PPD-B and PPD-A reactions of at least greater than 4 mm (B–A > 4 mm). PPD-A is used to control for the background sensitisation of animals with environmental mycobacteria to increase test specificity. To clear infection from herds, many countries use successive rounds of CCT testing, applying more stringent interpretation criteria by reducing the B–A cut-off value. For example, in the UK a so-called ‘severe interpretation’ of the test uses a B–A > 2 mm cut-off; in other countries a simple B > A interpretation is also used. Furthermore, some countries use yet another format of the tuberculin skin test to clear infected herds, the Single Cervical Tuberculin (SCT) test which relies on the injection of PPD-B only. However, due to its lower specificity compared to the CCT test, one limitation of using the SCT test to clear infected herds is the potential removal of more false-positive results. The SCT is also used as a trade (pre-export) test in the UK and Ireland, and as a primary bTB screening test for cattle in other European countries with active bTB surveillance programmes. Lastly, the site of tuberculin injection may vary, for example in New Zealand the screening test for bTB involves injection of PPD-B into the caudal folds (Caudal Fold Test).

Whilst it would be inappropriate to minimise the impact and critical importance of PPDs on the control and eradication of bTB in many countries, including the USA, New Zealand and many countries of the European Union, PPDs are not without a number of critical limitations. PPDs are crude extracts of mycobacterial culture supernatants that are difficult to standardise, and are largely undefined in their overall content and the identity of active components. The quality control processes, to determine the PPDs’ biological activity (potency), depend solely on the ‘guinea pig potency test’ that is notoriously unreliable and difficult to perform and to standardise. Furthermore, in the case of PPD-B, both production and potency testing require expensive biocontainment level 3 (BCL3) laboratory and animal housing facilities. In contrast, the use of defined antigens would have multiple advantages over tuberculin: definition of the active components; ease of manufacture; no BCL3 requirements—which would open the market to multiple producers, to generate competition and choice; and easier quality assurance, consistency and control of manufacture which would not be reliant on an antiquated bioassay but on chemical and biochemical techniques. An additional known limitation of tuberculin-based tests is that, depending on the test format, either their sensitivity or specificity can be impaired by infection with, or vaccination against, *M. avium* ssp. *paratuberculosis* (MAP), the causative agent of Johne’s Disease (JD)^10^.

Our work on the development of a DIVA skin test (DST) based on the three antigens ESAT-6, CFP-10 and Rv3615c has provided proof of concept that skin test reagents comprising defined protein antigens or synthetic peptides can be developed^11,12^. These formulations can also be applied to IGRA testing^11^. The DST was designed for use alongside BCG vaccination in cattle and was optimised to detect infected amongst vaccinated animals with optimal specificity matching that of the CCT. Building on the DST, the aim of the current study was to develop a molecularly defined tuberculin (MDT) skin test reagent that does not display the limitations of tuberculin PPD-based reagents described above. However, the scope of the application of this novel reagent is wider and not limited by the pre-condition of DIVA functionality as its application is aimed at testing animals that are not being BCG vaccinated, which will be the overall majority of cattle globally; whereas it is likely that cattle vaccination, if approved for use in the UK, will only be applied to particular herds or epidemiological scenarios. This lack of requirement for DIVA functionality has the potential to widen the repertoire of specific antigens that can be added to the three DST antigens in an extended cocktail. The specific aims of this project were therefore to develop a MDT reagent with increased signal strength compared to the DST, as well as test sensitivity similar to the SCT.

## Results

### Selection of antigens to be included in the MDT

To select additional antigens that could be added to the three DST antigens ESAT-6, CFP-10 and Rv3615c, we prepared a list of antigen candidates (Table 1). These proteins were selected because they showed promise, in earlier published and unpublished studies in our laboratory, of being specifically recognised in infected cattle but not in naïve cattle or those sensitised with environmental mycobacteria. However, they demonstrated no DIVA utility, as BCG vaccinated animals did recognise these proteins. Eighteen proteins were listed and prepared either as recombinant proteins or as a set of overlapping 20-mer peptides. To reduce the candidate pool, the antigens were applied in IGRAs using cryopreserved peripheral blood mononuclear cells (PBMC) from cattle naturally infected with *M. bovis*, or from animals free of bTB (14 animals each). Recombinant ESAT-6, CFP-10 and Rv3615c were also included in this screening exercise. As expected from testing samples from outbred populations, a wide range of responses were observed for the antigens (Table 1). Selection criteria for antigen inclusion into the MDT reagent were that they did not induce responses in the uninfected animals, but stimulated statistically significantly stronger responses in PBMC from infected animals compared to the uninfected controls. Four of the 18 antigens tested fulfilled these criteria (Rv1789, Rv3478, Rv3616c and Rv3810 in bold in Table 1). Furthermore, responses to ESAT-6, CFP-10 and Rv3615c were also stronger in PBMC from infected animals compared to uninfected controls. In addition to the four novel antigens, we also included into the MDT a previously described antigen, Rv3020c^13,14^. Thus, the composition of the MDT to be taken forward into the in vivo skin test and in vitro IGRAs described in the next paragraphs was as follows: Rv1789, Rv3020c, Rv3478, Rv3615c, Rv3616c, Rv3810, Rv3874 (CFP-10) and Rv3875 (ESAT-6). This prototype MDT was formulated as a cocktail of 7 recombinant proteins (Rv1789, Rv3020c, Rv3478, Rv3615c, Rv3810, Rv3874, and Rv3875) and one set of overlapping synthetic peptides representing Rv3616c (Supplemental Table 2). Responses induced by the MDT were compared to the DST cocktail of recombinant ESAT-6, CFP-10, and Rv3615c proteins.

**Table 1:** Summary of antigen details and PBMC IFN-γ responses.

*M. tb* designation (references)	Antigen format	Number of peptides	Control median ΔOD (min, max)	Infected median ΔOD (min, max)	*p* value
Rv0445c^(U)^	Peptide pool	17	0.049 (0.012, 0.422)	0.076 (− 0.125, 0.3420)	0.626
Rv0288^17^	Protein	N/A	0.002 (− 0.005, 0.085)	0.014 (− 0.005, 0.681)	0.382
Rv1038c^13^	Peptide pool	11	0.073 (0.016, 0.421)	0.106 (0.012, 0.403)	0.454
Rv1195^18^	Peptide pool	11	0.060 (0.010, 0.315)	0.111 (− 0.069, 0.315)	0.246
Rv1197^13^	Peptide pool	11	0.091 (0.010, 0.427)	0.121 (0.026, 0.439)	0.594
Rv1253^(U)^	Peptide pool	69	− 0.002 (− 0.011, 0.019)	0.007 (− 0.217, 0.139)	0.533
Rv1387^18^	Peptide pool	66	− 0.001 (− 0.005, 0.162)	0.027 (− 0.087, 0.163)	0.301
Rv1789^18^	**Peptide pool**	**48**	**0.001 (**− **0.006, 0.028)**	**0.037 (**− **0.003, 0.287)**	**0.001**
Rv1792^13^	Peptide pool	11	0.054 (0.008, 0.391)	0.110 (0.021, 0.420)	0.223
Rv1983^19^	Peptide pool	69	− 0.001 (− 0.011, 0.019)	− 0.003 (− 0.192, 0.141)	0.937
Rv2608^(U)^	Protein	N/A	0.116 (0.020, 0.968)	0.223 (0.012, 0.643)	0.427
Rv3017c^13^	Peptide pool	14	0.078 (0.000, 0.405)	0.100 (− 0.138, 0.235)	0.991
Rv3444c^13^	Peptide pool	11	0.052 (0.015, 0.360)	0.102 (− 0.157, 0.313)	0.571
Rv3478^(U)^	**Peptide pool**	**48**	− **0.002 (**− **0.007, 0.058)**	**0.064 (**− **0.165, 0.478)**	**0.018**
Rv3616c^20^	**Peptide pool**	**48**	**0.027 (**− **0.008, 0.181)**	**0.271 (0.063, 1.331)**	**< 0.0001**
Rv3810^(U)^	**Peptide pool**	**34**	**0.002 (**− **0.010, 0.038)**	**0.094 (**− **0.061, 0.390)**	**0.001**
Rv3872^19^	Protein	N/A	0.099 (0.011, 0.572)	0.081 (− 0.180, 0.473)	0.725
Rv3873^19^	Protein	N/A	0.096 (0.021, 0.637)	0.072 (− 0.168, 0.500)	0.812
Rv3874^21^ (CFP-10)	Protein	N/A	0.034 (0.004, 0.497)	0.385 (0.012, 1.355)	0.005
Rv3875^22^ (ESAT-6)	Protein	N/A	0.003 (− 0.007, 0.370)	0.221 (− 0.013, 1.460)	0.005
Rv3615c^23^ (EspC)	Protein	N/A	0.114 (0.035, 0.617)	0.201 (0.034, 0.596)	0.071

^(U)^Unpublished.

### Skin test responses to MDT

**Figure 1:**
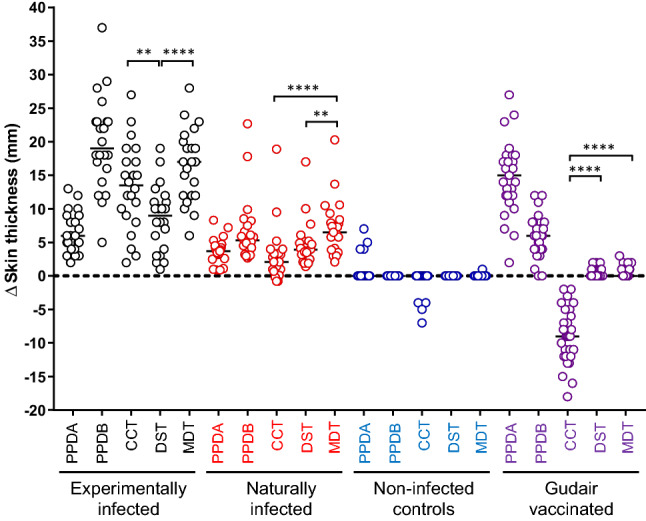
Comparison of MDT and DST induced skin test reactions in cattle. Skin test responses to PPDs, DST and MDT reagents were measured at 72 h after injection in cattle experimentally infected with *M. bovis* (*n* = 22), cattle naturally infected with *M. bovis* (n = 21), naive controls (*n* = 30) and Gudair vaccinates (n = 29). Results are expressed as the difference in skin thickness between the pre- and post-skin test readings. Each symbol represents an individual animal while horizontal lines represent group medians. **p < 0.01, ****p < 0.0001, Friedman test with Dunn’s multiple comparisons test.

In the next phase of this project, we undertook skin testing with the MDT reagent in four groups of animals: experimentally *M. bovis* infected cattle (n = 22), naturally *M. bovis* infected animals (n = 21), naive controls (n = 30) and a group of calves vaccinated against JD using the Gudair vaccine (n = 29). MDT, DST, PPD-B and PPD-A were injected in a Latin Square arrangement. The results are shown in Fig. 1. As expected strong PPD-B responses were observed in both groups of *M. bovis* infected animals resulting in a strongly PPD-B-biased CCT (PPD-B minus PPD-A) response (Fig. 1). Experimentally infected calves exhibited significantly stronger skin responses to MDT than to DST (*p* < 0.0001), and responses to DST were also significantly lower compared to the CCT (*p* = 0.0060). The significantly increased reactivity of MDT compared to DST was confirmed when naturally infected cattle were tested (Fig. 1, *p* = 0.0012). Interestingly, the MDT also induced significantly larger reactions than determined with the CCT (Fig. 1, *p* < 0.0001). The specificity of both DST and MDT were confirmed by their unresponsiveness in naïve cattle (Fig. 1). The effective sensitisation to MAP antigens following Gudair vaccination was demonstrated by the strong skin test responses observed following PPD-A, which also resulted in strongly PPD-A biased CCT responses (Fig. 1). DST and MDT, on the other hand, induced no or very low skin test reactions in Gudair vaccinated animals, thus further highlighting their specificity (Fig. 1). Therefore, we could demonstrate in this series of experiments that the MDT induced skin test responses of increased signal strength compared to the DST, at a comparably high specificity.

To assess whether the increased signal strength observed with MDT translated into increased sensitivity at more robust cut-off values compared to the DST, we applied a range of cut-off values to tabulate relative sensitivity and specificity of DST and MDT, and compared these values to the corresponding values obtained with CCT and SCT. As Table 2 shows, we assessed the effect of different DST and MDT cut-off values on sensitivity and specificity ranging from ≥ 2 mm (the cut-off commonly applied to the DST) to ≥ 5 mm. At all cut-offs applied, the MDT exhibited a greater sensitivity in experimentally and naturally infected animals compared to the DST, with this improved sensitivity becoming statistically significant at the ≥ 5 mm cut-off value (with relative sensitivities of 100% and 73% for the MDT and DST respectively in experimentally infected cattle, and 71% and 29% respectively in naturally infected cattle, *p* < 0.05 and *p* < 0.01, Table 2). In both groups of infected cattle, MDT sensitivities at all cut-offs applied matched those of the SCT with overlapping 95% CI (Table 2). In contrast, MDT sensitivities at all cut-off values applied were superior compared to CCT when interpreted at the > 4 mm and > 2 mm cut-offs in both infected animal groups (Table 2). Furthermore, when compared to CCT interpreted at the B > A cut-off, equivalent MDT sensitivities at all cut-off values applied were observed in experimentally infected cattle. Similar outcomes were observed in naturally infected cattle, with the exception that slightly lower sensitivities for MDT were observed at the higher cut-off values (≥ 4 and ≥ 5 mm) compared to the B > A interpretation of the CCT, although these did not achieve statistical significance. The relative sensitivities of the DST at the ≥ 2 and ≥ 3 mm cut-offs also matched CCT at the > 4 mm cut-off in experimentally infected animals, with the ≥ 2 mm DST cut-off matching relative sensitivities for the CCT at the > 2 mm interpretation, and only slightly lower than the CCT at the B > A interpretation, in this group of animals. In the group of naturally infected cattle, the DST at cut-offs up to ≥ 4 mm performed as well as, if not better, than the CCT interpreted at the > 4 and > 2 mm cut-offs, with the ≥ 2 mm DST cut-off almost matching the relative sensitivity for the CCT using the B > A interpretation.

**Table 2:** Comparison of skin test results at defined cut-off values.

Cut-off	% positive [95% CI] (number positive/total number)							
	Experimentally infected		Naturally infected		Controls		Gudair vaccinated	
	DST	MDT	DST	MDT	DST	MDT	DST	MDT
≥ 2 mm	95 [78, 100] (21/22)	100 [85, 100] (22/22)	81 [60, 92] (17/21)	100 [85, 100] (21/21)	0 [0, 11] (0/30)	0 [0, 11] (0/30)	10 [4, 26] (3/29)	14 [5, 31] (4/29)
≥ 3 mm	86 [67, 95] (19/22)	100 [85, 100] (22/22)	71 [50, 86] (15/21)	95 [77, 100] (20/21)	0 [0, 11] (0/30)	0 [0, 11] (0/30)	0 [0, 12] (0/29)	3 [0, 17] (1/29)
≥ 4 mm	77 [57, 90] (17/22)	100 [85, 100] (22/22)	48 [28, 68] (10/21)	76 [55, 89]* (16/21)	0 [0, 11] (0/30)	0 [0, 11] (0/30)	0 [0, 12] (0/29)	0 [0, 12] (0/29)
≥ 5 mm	73 [52, 87] (16/22)	100 [85, 100]* (22/22)	29 [14, 50] (6/21)	71 [50, 86]** (15/21)	0 [0, 11] (0/30)	0 [0, 11] (0/30)	0 [0, 12] (0/29)	0 [0, 12] (0/29)
SCT^a^ (≥ 4 mm)	100 [85, 100] (22/22)		76 [55, 89] (16/21)		0 [0, 11] (0/30)		79 [62, 90] (23/29)	
CCT^b^ (B–A > 4 mm)	86 [67, 95] (19/22)		14 [5, 35] (3/21)		0 [0, 11] (0/30)		0 [0, 12] (0//29)	
CCT^b^ (B–A > 2 mm)	95 [78, 100] (21/22)		52 [32, 72] (11/21)		0 [0, 11] (0/30)		0 [0, 12] (0/29)	
CCT^b^ (B > A)	100 [85, 100] (22/22)		86 [65, 95] (18/21)		0 [0, 11] (0/30)		0 [0, 12] (0/29)	

*DST* DIVA skin test, *MDT* molecularly defined tuberculin.

*p < 0.05, **p < 0.01 McNemar test (compared to DST).

^a^SCT, Single Cervical Tuberculin skin test interpretation (skin reaction increase ≥ 4 mm on PPD-B site).

^b^CCT, Comparative Cervical Tuberculin skin test interpretation (skin reaction increases for PPD-B minus PPD-A (B–A) at different cut-offs.

Relative specificity values at the same cut-off points described above were compared after considering the results obtained in naïve and Gudair vaccinated cattle (Table 2). In naïve control animals, no false positive responses were observed to DST and MDT at any cut-off applied, nor when applying SCT and CCT at any interpretation criteria (100% specificity, Table 2). Whilst low positive responses (2 mm) were observed in 3/29 Gudair vaccinated animals after DST injection, raising the DST cut-off point to ≥ 3 mm restored specificity in this group to 100% (Table 2). Similarly, MDT injection induced low skin responses in 4/29 and 1/29 animals at the ≥ 2 and ≥ 3 mm cut-off points respectively, whilst 100% specificity was restored by applying a ≥ 4 mm MDT cut-off point (Table 2). As expected from the high PPD-A biased CCT responses, all Gudair vaccinated animals tested negative in the CCT at all interpretations. However, the specificity of the SCT was severely compromised in the Gudair vaccinates of which 79% tested positive (21% specificity, Table 2). In conclusion, we demonstrated that the MDT allows for a more robust setting of cut-off points than is possible for the DST. This allowed us to match SCT performance in all animal groups (when using the ≥ 4 mm cut-off for MDT) and at least match and possibly surpass CCT performance when applying this MDT cut-off. Furthermore, MDT does not compromise specificity in Gudair vaccinated animals as was observed for the SCT, and presumably would also overcome the masking effect of Gudair vaccinated or MAP infected animals that are also infected with *M. bovis*, which occurs when using the CCT in such dually infected animals due to high PPD-A responses.

### Application of the MDT in IGRAs

**Figure 2:**
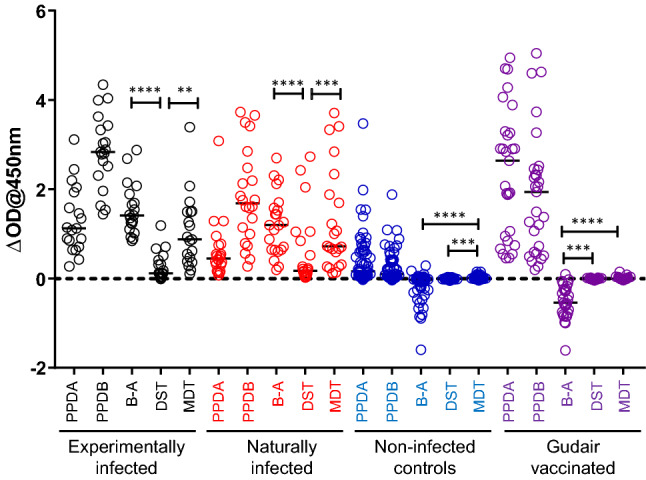
Quantification of MDT and DST induced in vitro IFN-γ production. Blood samples from cattle experimentally infected with *M. bovis* (*n* = 22), cattle naturally infected with *M. bovis* (n = 21), naive controls (*n* = 59) and Gudair vaccinates (n = 29) were stimulated in vitro with PPDs, DST and MDT reagents and IFN-γ production measured by ELISA. Each symbol represents an individual animal while horizontal lines represent group medians. **p < 0.01, ***p < 0.001, ****p < 0.0001, Friedman test with Dunn’s multiple comparisons test.

The IGRA is a valuable additional ancillary surveillance test applied alongside skin testing to maximise the detection of infected animals. Therefore, it was relevant to investigate MDT performance in this test format. Samples were obtained from the same animals as described above (22 experimentally infected, 21 naturally infected cattle, 29 Gudair vaccinated calves and an increased group of 59 naïve animals). Blood samples were taken prior to skin test antigen injection and stimulated in vitro with MDT, DST, PPD-A and PPD-B. In a pilot experiment, we had determined that DST and MDT performed optimally in terms of sensitivity and specificity at an assay concentration of 0.1 μg/ml. PPD-A and PPD-B were used at the concentrations they are applied in the GB routine surveillance programme (250 and 300 IU/ml, respectively). The results of this analysis is shown in Fig. 2. As in the case of the skin test responses presented in the previous paragraph, MDT induced significantly higher levels of IFN-γ in experimentally and naturally infected cattle compared to DST stimulation (Fig. 2. *p* = 0.0020 and *p* < 0.0001, respectively). The conventional interpretation of the IGRA is based on a cut-off calculated by subtracting PPD-A induced IFN-γ from IFN-γ induced by PPD-B stimulation (B–A). It is encouraging to note that whilst DST-induced responses in both categories of infected cattle were significantly lower than the B–A values (Fig. 2, *p* < 0.0001 for experimentally and naturally infected animals), the MDT-induced IFN-γ responses were not significantly different, again confirming that MDT is a more potent inducer of IFN-γ in infected animals than the DST (Fig. 2). Both the non-infected control animals and the Gudair vaccinates displayed strong PPD-A responses which resulted in PPD-A-biased B–A responses (Fig. 2). In contrast, neither the DST nor the MDT stimulated samples from Gudair vaccinated or uninfected control animals resulted in IFN-γ responses above the background of cultures without antigen (Fig. 2).

We next interpreted these data using as cut-off for positivity an antigen minus nil antigen IFN-γ optical density value at 450 nm of > 0.1. This is the cut-off used in routine GB IGRA surveillance testing for both B–A and ESAT-6/CFP-10 responses. The results of this analysis over the four groups of animals in respect to relative sensitivity and specificity are shown in Table 3. Paralleling the skin test results, MDT was able to detect both naturally and experimentally *M. bovis* infected cattle with high relative sensitivity (100% of animals in both groups, Table 3) matching the performance of the B–A read-out in these cohorts. In contrast, the DST detected a significantly smaller proportion of infected animals (63% and 68% of experimentally or naturally infected animals respectively, differences in both groups were significant with *p* < 0.05, McNemar’s test, Table 3). Both DST and MDT displayed high specificities (97–100%) comparable to those observed with avian and bovine PPD (B–A) in the two uninfected groups (non-infected controls and Gudair vaccinates, Table 3). In summary, these data also demonstrate the superior performance of the MDT antigens when applied in the IGRA. Furthermore, these data also clearly demonstrate that the MDT based IGRA testing could be used without interference from Gudair vaccination to serve as an ancillary test to skin testing in an effort to maximise the detection of bTB animals during herd clean-up.

**Table 3:** IGRA^a^ test results for DST and MDT reagents.

Animal group	Test performance	DST^b^	MDT^b^	B–A^c^
Experimentally infected (n = 19)	% Sensitivity [95% CI]	63 [41, 81]	100 [83, 100]*	100 [83, 100]
Naturally infected (n = 22)	% Sensitivity [95% CI]	68 [47, 84]	100 [85, 100]*	100 [85, 100]
Non-infected controls (n = 59)	% Specificity [95% CI]	100 [94, 100]	97 [88, 99]	97 [88, 99]
Gudair vaccinated (n = 29)	% Specificity [95% CI]	100 [88, 100]	97 [83, 100]	100 [88,100]

*p < 0.05, McNemar’s test (compared to DST).

^a^IGRA, interferon-gamma release assay using the BOVIGAM IFN-γ EIA kit.

^b^DST (DIVA Skin Test) or MDT (Molecularly Defined Tuberculin) induced IFN-γ production. Cut-off for positivity = OD450 nm with MDT or DST minus nil antigen control > 0.1.

^c^B–A, comparison of IFN-γ induced by stimulation with PPD-B and PPD-A. Cut-off for positivity = OD450 for PPD-B minus PPD-A > 0.1.

## Discussion

The conventional strategy applied to control and eradicate bTB in cattle is based on the systematic tuberculin skin testing of herds and the slaughter of test-positive animals. This ‘test and slaughter’ approach has led to the eradication of bTB in many countries, particularly in high income ones such as the USA, Canada, Australia and many European countries that can afford the application of such strategies. The mainstay of this strategy is the tuberculin skin test that since the 1930s has relied on the use of PPDs in a number of test formats. Whilst PPDs have been unquestionably highly successful test reagents, they suffer from a number of limitations, including potency determination based on a bioassay (Guinea pig potency assay) that, in of itself, also requires reproducible and high quality reference standards. As a consequence of these limitations alone, PPDs of vastly different quality are being used worldwide^15^. To overcome this and other limitations of PPDs, we embarked on developing a synthetic, MDT reagent composed of a small number of recombinant proteins and synthetic peptides. In this setting, MDT quality control and assurance would not depend on a bioassay but instead on biochemical and chemical methods of protein and peptide quantification and purity assessment. In addition, its immunological activity would be based on defined protein and peptide concentrations and not on units in relation to a standard.

An additional requirement to develop the MDT was that its specificity was not compromised in cattle sensitised, by infection or vaccination, against *M. avium* ssp *paratuberculosis* antigens. The data presented (Figs. 1, 2; Tables 2, 3) demonstrated the MDT’s specificity in MAP (Gudair) vaccinated cattle. Furthermore, we investigated MDT skin test reactions in an additional group of 30 Gudair vaccinated cattle (data not shown). As skin testing with the DST reagent was not performed in these additional animals, we have not included this data in Fig. 1 or Table 2. For these additional animals, no MDT positive skin test reactions were observed using the ≥ 3 mm cut-off point. Thus, the inclusion of these additional data improved our precision in the specificity estimate of the MDT in Gudair vaccinated animals by reducing the 95% CI for this estimate by almost a half at both the ≥ 3 mm and ≥ 4 mm cut-off points (98% [95% CI 91, 100] and 100% [95% CI, 94, 100] respectively). Thus, we have demonstrated that the MDT overcomes the specificity limitations of the SCT in MAP vaccinated, and presumably MAP infected cattle, which can result in a large proportion of false-positive reactions in these animal categories (see Table 2). Furthermore, whilst not directly tested in this study, it is also highly plausible that the MDT will overcome the sensitivity issues associated with the CCT in herds dually infected with MAP and *M. bovis,* which can result in false CCT-negatives due to the masking of PPD-B biased responses by high PPD-A skin reactions in MAP vaccinated or MAP infected cattle.

The approach we took began with a long list of potential antigens that we had identified in earlier studies as immunogenic in *M. bovis* infected cattle, but not recognised in uninfected animals. These antigens did not have a BCG DIVA utility as they were also recognised in BCG vaccinated animals. This outcome was confirmed in an experiment in which each of the 10 BCG vaccinated calves responded with skin test reaction after injection of MDT (data not shown). However, there is also a strong desire to develop a defined reagent that can match SCT sensitivity in unvaccinated cattle with a more robust cut-off for positivity in skin testing operations than can at present be realised with the existing DST antigens. Therefore, we compared the performance of MDT with that of the DST composed of ESAT-6, CFP-10 and Rv3615c. Our data provide proof of concept that the MDT has the potential to fulfil these criteria. Another requirement of the MDT was that it matches the specificity of tuberculin based tests (i.e. CCT and SCT) as well as the high specificity established for the DST. Again, our data support the conclusion that the MDT cut-offs can be adjusted to match these requirements, even in a very challenging group of cattle that were highly sensitised to MAP antigens after Gudair vaccination.

The first task was to reduce the list of 18 proteins to a shortlist of proteins to be included in the MDT. For this screening test we used IGRA responses induced in PBMC from infected and uninfected animals. We accepted that using IGRA as a ‘gating in’ test for skin test antigens would have potential drawbacks, but for practical reasons it was impossible to screen such a large number of antigens (including PPD-A, PPD-B and the DST) using in vivo skin tests. We were also confident that IGRA would be a convenient substitute to in vivo screening as in previous studies we demonstrated that antigens inducing skin test responses were also able to induce IGRA responses. In addition, antigens such as CFP-10 and ESAT-6, which are being employed in human IGRA-based diagnosis, are also potent human skin test antigens. The simple ‘gating in’ criteria we used in the antigen selection phase of the project (no IGRA responses in uninfected animals and statistically significantly higher IFN-γ responsiveness in infected cattle) were successful in down-selecting antigens for inclusion in the MDT. We also noted that PBMC from 7/14 infected cattle used in the screening process did not respond to ESAT-6. The four antigens prioritised by our approach were recognised by between one and five of these ESAT-6-negative animals demonstrating their potential to complement the responses to the DST antigens to increase overall signal strength and sensitivity.

The five antigens that were added to ESAT-6, CFP-10 and Rv3615c to make up the MDT all belong to protein families previously highlighted to contain immune-dominant antigens recognised by T-cells from tuberculous humans and cattle. These included the ESAT-6 family (i.e. Rv3020c); the PPE family (i.e. Rv1789 [PPE26] and Rv3478 [PPE60]), and a cell-surface protein (i.e. Rv3810 [PirG]). In addition, Rv3616c (ESPA) is part of the esx-1 secretion system, and like Rv3615c, involved in the secretion of ESAT-6 and CFP-10. Our intention was to present the prototype MDT used in this study as a formulation of individual recombinant proteins and compare it to a DST cocktail of three proteins. We were able to procure such recombinant proteins for all antigens apart from Rv3616c, which was therefore represented in the MDT as a cocktail of 20 synthetic peptides covering its complete sequence. We had originally shown that short synthetic peptides (16 to 20-mers) can be used to skin test cattle although signal strengths were inferior to protein-based skin test reagents. However, in a recent study^11^ we used longer peptides (40-mers) to represent the DST antigens ESAT-6, CFP-10, and Rv3615c and could demonstrate equivalent skin test sensitivity compared to a recombinant DST fusion protein. Although we were unable to perform a head-to-head comparison of only the Rv3616c peptide cocktails of 20-mer and 40-mer peptides as skin test reagents, we compared MDT preparations containing either 20-mer or 40-mer Rv3616c peptide cocktails in infected cattle. This comparison revealed that the MDT containing 40-mer peptides induced significantly stronger skin test responses than the MDT preparation containing the 20-mer peptides (data not shown). The mechanisms underlying the observation that longer peptides are more effective in the skin test than shorter peptides has not been formally investigated. However, it is likely that longer peptides are processed within antigen presenting cells and therefore are more effectively loaded onto MHC class II molecules. Shorter peptides, on the other hand, are probably loaded onto the MHC molecules by the less effective process of replacing the cell surface peptides already complexed to MHC proteins.

It is interesting to note the differences in the responses between experimentally and naturally infected cattle. Responses in experimentally infected animals are usually higher than those of naturally infected cattle, so the results in this respect are not surprising. However, it was surprising to observe that only 14% of the naturally infected animals tested positive to the standard CCT despite them being selected for inclusion into the study based on a positive disposing CCT. However, these animals at the time of testing the MDT had undergone between 2 and 4 previous CCT. We have shown previously that repeated skin testing of CCT reactor animals can lead to a gradual decrease of CCT responsiveness^16^ and this might be the case here. Interestingly, the MDT responsiveness seemed not to be, or only marginally, affected as we observed strong responses in these naturally infected cattle to this reagent.

In conclusion, the MDT promises to be a robust diagnostic skin and blood test reagent capable of addressing some of the limitations of the PPDs. Moreover, its formulation might be refined to allow easier, and therefore, more cost-effective production.

## Materials and methods

### Preparation of antigens

#### In vitro assays

Of the 18 candidate antigens to be screened in the PBMC assay, four (Rv0288, Rv2608, Rv3872 and Rv3873) were sourced as recombinant proteins from a commercial manufacturer (Lionex Ltd, Germany) and used to stimulate cattle PBMC at a final concentration of 5 µg/ml. The remaining antigens (Rv0445c, Rv1038c, Rv1195, Rv1197, Rv1253, Rv1387, Rv1789, Rv1792, Rv1983, Rv3017c, Rv3444c, Rv3478, Rv3616c, and Rv3810) were prepared as 14 separate pools of overlapping synthetic peptides (20-mers overlapping by 12 amino acids; JPT Peptide Technologies, Germany). Details of the peptide pools are shown in Supplemental Table 1. The lyophilized peptide pools were reconstituted in RPMI 1640 (Gibco Life Technologies, UK) containing 2.25% DMSO to obtain a concentration of 55 µg of each peptide/ml, with the exception of Rv3616c which was reconstituted in RPMI 1640 containing 25% DMSO to obtain a concentration of 1 mg of each peptide/ml. All peptide pools were used to stimulate cattle PBMC at a final concentration of 5 µg of each peptide/ml. The individual DIVA recombinant proteins ESAT-6, CFP-10 and Rv3615c (Lionex Ltd) were also used at a final concentration of 5 µg/ml.

#### In vivo skin testing

ESAT-6, CFP-10, Rv1789, Rv3020c, Rv3478, Rv3615c and Rv3810 were sourced as recombinant proteins from a commercial manufacturer (Lionex Ltd). Rv3616c was prepared as a synthetic peptide pool consisting of sixteen 40-mers, three 25-mers and one 20-mer (GenScript Biotech, Netherlands) where each individual lyophilized peptide was first reconstituted in PBS to a concentration of 10 mg/ml and then combined together to obtain a peptide pool of 0.5 mg of each peptide/ml. Details of the peptide pools are shown in Supplemental Table 2. The MDT skin test reagent was then formulated by combining ESAT-6, CFP-10, Rv1789, Rv3020c, Rv3478, Rv3615c and Rv3810 proteins with the Rv3616c peptide pool so that each protein or individual peptide was at a concentration of 100 µg/ml. As a control, a skin test reagent (DST) comprised of ESAT-6, CFP-10 and Rv3615c proteins only was also formulated at 100 µg of each protein/ml. Bovine tuberculin (PPD-B) and avian tuberculin (PPD-A) were obtained from a commercial manufacturer (Thermo Fisher, UK).

### Animals

For the initial in vitro antigen screening, archived PBMC from the following groups of cattle (*Bos taurus taurus*) were used: (1) naturally *M. bovis*-infected cattle originating from UK herds known to have bTB (natural infection was confirmed by post mortem and/or culture analysis); and (2) non-infected control cattle originating from UK herds in the Low Risk Area of England that were Officially TB Free for over 5 years. For in vivo testing of skin test reagents, the following groups of cattle were used: (1) experimentally *M. bovis* infected cattle consisting of male calves experimentally infected with approx. 10,000 CFU of a field strain of *M. bovis* (AF2122/97) via the endobronchial route (infection was confirmed by post mortem and/or culture analysis); (2) naturally *M. bovis* infected cattle sourced from TB breakdown herds in Ireland, consisting of castrated males from Holstein–Friesians or diary cross breeds that had tested positive in the CCT test at standard interpretation and were also test positive in the IGRA; (3) non-infected control calves (as described above); and (4) Gudair vaccinated calves (5–7 months old, males, Holstein–Friesian breed or crosses thereof) that were vaccinated with 1 ml Gudair vaccine (Virbac Ltd, UK) via the subcutaneous route. The experimentally *M. bovis* infected calves were skin tested five weeks post infection while the Gudair vaccinated calves were skin tested 8 weeks post vaccination. The same four groups of cattle were also used to provide blood samples for the in vitro whole blood assay prior to skin testing. For the experimentally *M. bovis* infected calves and the Gudair vaccinated calves, these were obtained five weeks post infection and 8 weeks post vaccination, respectively. All animal procedures at APHA were approved by the APHA Animal Welfare and Ethical Review Board (Home Office Licence Number: PF7D840A5), and those in Ireland were approved under a research licence issued by the Health Products Regulatory Agency (Project Authorisation Number: AE19113/P008). All animal experiments were performed in compliance with ARRIVE guidelines and in accordance with Home Office and local AWERB guidelines and regulations.

### In vitro stimulation of PBMC

PBMCs were isolated from heparinised cattle blood by density gradient centrifugation using Histopaque 1077 (Sigma-Aldrich, UK) and cryopreserved in foetal calf serum (Sigma-Aldrich, UK) containing 10% DMSO (Sigma-Aldrich, UK) prior to use. Cryopreserved PBMC were thawed as quickly as possible in a water bath at 37 °C before adding complete medium [RPMI 1640 containing 2 mM GlutaMax, 25 mM HEPES, 0.1 mM NEAA, 5 × 10^−5^ M β-mercaptoethanol, 100 U/ml penicillin, 100 µg/ml streptomycin (Gibco Life Technologies, UK) and 10% foetal calf serum (Sigma-Aldrich, UK)] in a dropwise manner. After centrifugation at 350 g for 10 min at room temperature, the supernatant was discarded, the cell pellet gently loosened and re-suspended in complete medium and the cells counted using a haemocytometer. PBMCs were plated at 2 × 10^5^ cells/well in duplicate wells of a 96-well plate and stimulated with and without antigens at a final volume of 275 µl/well for 3 days at 37 °C in the presence of 5% CO_2_, following which cell supernatants were removed and stored at − 80 °C until required.

### In vitro stimulation of whole blood

Heparinised blood samples from all groups of animals were stored overnight at room temperature before stimulation (250 µl in duplicate wells of a 96-well plate) for 20 to 24 h at 37 °C in 5% CO_2_ with MDT and DST reagents at a final assay concentration of 0.1 µg/ml for each protein/peptide component. As positive and negative controls, blood samples were cultured with pokeweed mitogen (10 µg/ml; Sigma, UK) and RPMI-1640 alone (Gibco, UK) respectively. After stimulation, blood was centrifuged at 300 g for 10 min and the plasma supernatant was harvested and stored at − 80 °C until required.

### IGRA

IFN-γ in plasma and PBMC culture supernatants was quantified using the commercially available BOVIGAM enzyme-linked immunosorbent assay (ELISA) kit (Thermo Fisher Scientific, USA). Results were expressed as the optical density at 450 nm (OD_450_) for cultures stimulated with antigen minus the OD_450_ for cultures without antigen (i.e. ΔOD_450_).

### Skin test procedure

Injection sites located in the border of the anterior and middle third of the neck on either side of the cow were clipped and skin thickness recorded. PPD-A and PPD-B were administered in a 0.1 ml volume via intradermal injection as per the manufacturer’s recommendations. DST and MDT reagents were administered in a similar manner so that each individual protein or peptide was delivered at a 10 µg dose. To account for potential injection site differences, a Latin Square design was applied with animals randomly assigned to the Latin Square combinations; the operators were blinded to the nature of the injection solutions. Skin thickness was measured again by the same operator 72 h after administration, and the difference in skin thickness (mm) between the pre- and post-skin test readings recorded.

### Statistical analysis

All statistical analyses were performed using Prism 7 (GraphPad Software, USA). PBMC IGRA responses were compared using the Mann–Whitney U test. The McNemar matched pair test was used to compare proportions of test positives and negatives. Comparisons between the skin test responses or the whole blood IGRA responses induced with the antigens were analysed using the Friedman test with Dunn’s multiple comparisons test.

## Supplementary Information

Supplementary Tables.
